# Risk of childhood trauma exposure and severity of bipolar disorder in Colombia

**DOI:** 10.1186/s40345-023-00289-5

**Published:** 2023-02-10

**Authors:** Hernán Guillen-Burgos, Sergio Moreno-Lopez, Kaleb Acevedo-Vergara, Manuel Pérez-Florez, Catherine Pachón-Garcia, Juan Francisco Gálvez-Flórez

**Affiliations:** 1 Center for Clinical and Translational Research, La Misericordia Clinica Internacional, Barranquilla, Colombia; 2 School of Health Science, Universidad Simon Bolivar, Carrera 54 No 64-222, Barranquilla, Colombia; 3 School of Medicine, Universidad de Los Andes, Bogotá, Colombia; 4 Zerenia Clinics, Bogotá, Colombia; 5 Latin American Society of Liaison Psychiatry (SOLAPSIQUE), Bogotá, Colombia; 6 Otolaryngology and Allergology Research Group, Unidad Médico Quirúrgica de Otorrinolaringología (UNIMEQ‐ORL), Bogotá, Colombia; 7 Instituto Cardiovascular del Cesar, Valledupar, Colombia

**Keywords:** Case–control study, Childhood trauma, Traumatic life events, Severe bipolar disorder

## Abstract

**Background:**

Bipolar disorder (BD) is higher in developing countries. Childhood trauma exposure is a common environmental risk factor in Colombia and might be associated with a more severe course of bipolar disorder in Low-Middle Income-Countries. We carried out the first case–control study in Colombia using a structural clinical interview and the Childhood Trauma Questionnaire-Short Form (CTQ-SF) to describe the prevalence and association between trauma exposure during childhood with a severe course of illness (early age onset, rapid cycling, ideation or suicide attempt, or ≥ 3 hospitalization) in a sample of BD patients.

**Results:**

A total of 114 cases and 191 controls evaluated showed the following results. Cases included 61.4% BD type I and 38.6% BD type II. The median age was 31.5 years (IQR, 75–24) for BD patients and 31 years old (IQR, 38–24) for healthy controls. A higher prevalence of childhood trauma was evidenced in cases compared to controls. Emotional abuse, physical abuse, sexual abuse, physical neglect and emotional neglect evidenced a strong association with severe bipolar disorder (*OR* = 3.42, p < .001; *OR* = 4.68, p < .001; *OR* = 4.30, p = .003; *OR* = 5.10, p < .001; *OR* = 5.64, p < .001, respectively).

**Conclusions:**

This is the first association study between childhood trauma exposure as a higher risk for a severe course of illness in BD patients in Colombian. Our findings highlight the higher prevalence of childhood trauma in bipolar patients and the strong association of childhood trauma with severe bipolar disorder. These findings are relevant for screening and evaluating childhood trauma exposure during the course of BD patients.

## Background

Bipolar disorder (BD) is a common and severe mental disorder with an estimated prevalence of 3% worldwide (Ketter 2010; Kessler et al. 2005). Bipolar disorder`s etiology remains still to be unclear. However, numerous genetic (Gordovez and McMahon 2020), biological (Nurnberger et al. 2014), clinical (Grande et al. 2016), and environmental (Aldinger and Schulze 2017) factors have been associated with the time of onset as well as a severe course of illness. Theories of neuro-progression of bipolar disorder are related to a more severe course of the disease and have been associated with an earlier age of onset (Yatham et al. 2018), higher rates of suicidal ideation or attempts (Latalova et al. 2014), development of rapid cycling (Fountoulakis et al. 2013; Galvez et al. 2014), high recurrence rates (Yatham et al. 2018), and frequent in-patient treatment in psychiatric units (Hassan and Lage 2009).

The lifespan of traumatic events and childhood trauma (CT) as environmental factors have been associated with the onset and severity of psychiatric disorders (Aldinger and Schulze 2017; McLaughlin et al. 2010; McKay et al. 2021; Etain and Aas 2021). In bipolar patients, psychosocial stressors, trauma life events, and CT have been identified during the last twenty-year as risk factors in their development and course of illness (Fisher and Hosang 2010; Samuel and Soares 2000; Aas et al. 2016a), however, the studies have evidenced several limitations such as small samples, absence of structured clinical interview for diagnoses, lack of use of standardized childhood trauma assessments, and the non-use at-recruitment of assessments on the current state of mood (Etain and Aas 2021; Daruy-Filho et al. 2011). Besides, the majority of these studies have been conducted in high-income countries (HICs), with little attention in low-and middle-income countries (LMICs) where more and high-quality research is needed as previously mentioned by World Health Organization (WHO) and global mental health researchers (Saxena et al. 2013; Lund et al. 2016; Patel et al. 2018; Patel 2007).

In this sense, it is important to identify the prevalence and association of childhood trauma and its influence on the development and course of bipolar illness in LMCIs, specifically with a sample of bipolar patients in Colombia. Childhood trauma have not been explored enough in patients with mental disorders in Colombia (Gomez-Restrepo et al. 2018; Castañeda-Polanco and Camargo-Barreto 2018; Arenales et al. 2008). Moreover, there are no studies exploring childhood trauma in the course of illness for bipolar patients in Colombia. Thus, the aims of this study are to describe the prevalence of childhood trauma in bipolar patients and the association between exposure to childhood trauma with regard to the severity of bipolar disorder patients in Colombia.

## Methods

### Participants and procedure

A case–control study that assessed outpatients between 18 and 65 years old, at a teaching hospital [removed for blind review] in Barranquilla, Colombia was carried-out. The study followed the STROBE guidelines (Elm et al. 2014) to improve the quality of research conducted in human subjects. All participants in the cases group were recruited from the outpatient mental health clinic program of the teaching hospital and also referred from private consultations. The controls were recruited from the Healthy Volunteers Program from [removed for blind review]. Moreover, all subjects were assessed for diagnosis with the Diagnostic Statistical Manual for mental disorders (DSM-5) (American Psychiatric Association 2013). Furthermore, clinical diagnoses for bipolar disorder were confirmed using the Structured Clinical Interview for DSM-5 Clinician Version (SCID-5-CV) (First et al. 2016) conducted by trained mental health professionals.

Our inclusion criteria limited the sample of bipolar patients to subjects with: (i) ages between 18 and 65 years old; (ii) minimal levels of literacy of participants (reading and writing); (iii) participants receiving treatment with mood stabilizers for at least six months before the enrollment in the study. On the other hand, the inclusion criteria for control subjects were: (i) ages between 18 and 65 years old; (ii) not having a diagnosis of mental, cognitive, or behavioral disorders; (iii) absence of past psychiatric history of manic, hypomanic or mixed episodes; (iv) absence of other types of mood episodes such as post-partum depression, major depressive disorder; or comorbid depression due to a medical condition, organic affective disorders, substance/medication-induced or; (v) no history of psychotic episodes; (vi) not having a family history of mood disorders and psychotic disorders in first-degree relatives; (vii) no history of suicidal ideation or suicide attempts.

Exclusion criteria limited to: (i) intellectual disability; (ii) Young Mania Rating Scale ≥ 12; (iii) Bipolar Depression Rating Scale > 3; (iv) lifetime history of mental, cognitive or behavioral disorder; (v) lifetime history of neurological disorder; (vi) Bipolar disorder induced by substance/medication-induced or due to another medical condition.

The study was approved by Ethics Committee for the [removed for blind review] in Barranquilla, Colombia. This study was conducted in accordance with the ethical considerations of the Declaration of Helsinki and Good Clinical Practices. All participants signed an informed consent to participate in the research.

### Measures

All participants were assessed with a structured clinical interview including demographic, clinical and psychometric instruments allocated in the REDcap software (Licensed by Vanderbilt University) following a strict protocol of clinical assessment. Three certified mental health professionals assessed all the participants. Moreover, all cases and controls were revised and confirmed by a senior psychiatrist with training and vast experience in the diagnosis and treatment of mood disorders. Furthermore, all prevalent cases with a diagnosis of bipolar disorder between 2017 and 2021 were selected.

All participants were assessed with the SCID-5-CV, the Young Mania Rating Scale (YMRS), and the Bipolar Depression Rating Scale (BDRS). Additionally, exposure to childhood trauma was assessed using The Childhood Trauma Questionnaire-Short Form (CTQ-SF). The CTQ-SF is a brevity 28-item Likert-type, with a five-factor structure: emotional abuse (EA), physical abuse (PA), sexual abuse (SA), physical neglect (PN), and emotional neglect (EN), self-administered instrument in order to assess multiple types of trauma during childhood (29). In the past, the CTQ-SF has shown adequate psychometric properties to be included as an objective measurement of childhood trauma (Bernstein et al. 2003). Thus, the cut-off score (moderate to severe) for each subtype of childhood trauma included in the CTQ-SF has been described in a previous study to dichotomize each sub-score and classify subjects as having/not having a history of childhood trauma as suggested by Bernstein & Fink (≥ 13 EA; ≥ 10 PA; ≥ 8 SA; ≥ 15 EN; ≥ 10 PN) (Bernstein and Fink 1998). Additionally, we assessed the sample for probable Post-traumatic Stress Disorder symptoms, as a controlled variable. We used the Life Events Checklist for DMS-5 (LEC-5) and the Post-traumatic Stress Disorder Check-list for DSM-5 (PCL-5) to identify possible cases of PTSD symptoms. The PCL-5 is a self-reported 20-item PTSD Check-list for DSM-5 with a cutoff score ≥ 33 to be able to screen for probable PTSD (Blevins et al. 2015). We also performed the Alcohol Use Disorders Identification Test (AUDIT) which is a 10-item screening tool developed by World Health Organization (WHO) in order to control for possible confounders related to harm associated with alcohol consumption with inadequate detection (AUDIT 2022). All these instruments were performed implementing their respective validated Spanish versions.

Demographic and clinical factors were collected through (i) a semi-structured interview collected in REDcap, and (ii) through the Electronic Health Records including those years in which follow-up for cases and controls were determined (2017–2021).

### Outcomes

We generate an outcome variable named severe bipolar disorder defined by course severe of bipolar disorder as the presence of any clinical indicator of severity, previously delimited by the research team (early-onset, rapid cycling, ideation or suicide attempt, or ≥ 3 hospitalizations per year) based on the available evidence on previously reported markers of severity associated with the clinical course (Galvez et al. 2014; Leboyer et al. 2007; Garno et al. 2005; Leverich and Post 2006; Cate Carter et al. 2003; Suominen et al. 2007; Brown et al. 2015; Leverich et al. 2002; Carballo et al. 2008; Hamilton et al. 2016). Also, we carried out bivariate and regression analyses with each clinical indicator of severity as an outcome.

### Data analysis

Statistical analysis was performed with StataSE® v17 software. Both absolute and relative frequencies were calculated along with percentages for the qualitative variables. We also performed the mean and median included for the measures of central tendency, while measures of dispersion the standard deviation and interquartile range (IQR) were included for quantitative variables, the above conditioned to the normality of the variables. Additionally, a bivariate analysis was performed for categorical variables with chi-square or exact fisher test. Explanatory variables related to Childhood trauma were derived from CTQ-SF subcategories. We dichotomize each subcategory according to the cutoff point described above for each subcategory. Furthermore, a robust logistic regression models were used to estimate the outcomes of interest controlling for possible confounding covariates or others effect modifiers. The purpose of these models was to describe the association between trauma exposure in childhood and the degree of severity of bipolar disorder, adjusted by sociodemographic and other factors such as probable PTSD symptoms and symptoms related to Alcohol Use Disorder. Relationships between exposure and outcome were considered for inclusion in the model that resulted at a statistical level with a p-value in the association tests with a cutoff point of 0.157 based on the suggestions of Heinze and Dunkler (2017). Thus, variables that presented a positive association with the exposure and the outcome were considered as possible confounders. Nevertheless, variables that were also determined to be relevant from the clinical and biological plausibility were also added. The performance of the models will be compared using the Akaike criteria (AIC) and the Bayesian information criteria (BIC). A level of significance of 5% will be carried out for the analysis, estimates and clinical description in this case–control study. Model assumptions were validated through a linearity test, the Hosmer–Lemeshow test, an estimation of deviance residuals and Cook’s values, and a comparison between the crude and the adjusted models.

Regarding outcomes of clinical severity, we generate a dichotomized variable named “severe bipolar disorder” to classify subjects as having/not having a severe illness. Any patient that presented any of the four positive clinical indicators of severity stipulated for our case – control study which included and were limited to age of onset < 25 years, suicidal ideation or suicide attempts, rapid cycling course of illness (at least 4 mood episodes (mania, hypomania, or depression) in the previous 12 months), ≥ 3 inpatient treatment per year in psychiatric units for manic, hypomanic, mixed and depressive mood episodes were considered having severe illness. Also, we carried out bivariate and regression analyses with each clinical indicator of severity assessed as an outcome.

## Results

### Demographic and clinical characteristics

We included 114 cases and 191 controls, for a total of 305 study participants in the study. Among these 114 participants who had a positive and confirmed diagnosis of BD (34 men and 80 women), 61.4% (70/114) had a diagnosis of BD I and 38.6% (44/114) had a diagnosis of BD II. According to childhood trauma subcategories, the prevalence were 31.58% (36/114) EA, 33.33% (38/114) PA, 31.58% (36/114) SA, 21.93% (25/114) PN, 30.70% (35/114) EN. Compared to the control group, the prevalence were 10.99% (21/191) EA, 8.38% (16/191) PA, 5.24% (10/191) SA, 5.76% (11/191) PN, 7.33% (14/191) EN (Table 1). Also, the 89.47% (102/114) of cases met criteria for severe illness according to the clinical indicators established (Table 2).

**Table 1:** Demographic characteristics of cases and controls (N = 305)

	Cases n (%)	Controls n (%)	Total n (%)	p value
No participants	114 (37.37)	191 (62.63)	305 (100)	
Age (Mdn, IQR)	31.5 (37–24)	31 (38–24)	31 (27–24)	0.850
Age by groups, n (%)				
18–25	36 (38.30)	58 (61.70)	94 (100)	0.029
26–49	76 (36.71)	131 (63.29)	207 (100)	
≥ 50	2 (50.00)	2 (50.00)	4 (100)	
Gender, n (%)				
Male	34 (31.48)	74 (68.52)	108 (100)	0.110
Female	80 (40.61)	117 (59.39)	197 (100)	
Marital status, n (%)				
Single	63 (33.87)	123 (66.13)	186 (100)	0.200
Married	39 (46.43)	45 (53.57)	84 (100)	
Divorced	12 (34.29)	23 (65.71)	35 (100)	
Ethnicity, n (%)				
White/hispanic	95 (36.54)	165 (63.46)	260 (100)	0.460
Black/Afro-descendent	19 (42.22)	26 (57.78)	45 (100)	
Education, n (%)				
Elementary	3 (42.86)	4 (57.14)	7 (100)	0.280
High School	59 (39.86)	88 (60.14)	148 (100)	
Technician	42 (35.59)	75 (64.41)	118 (100)	
Graduate/Postgraduate	10 (31.25)	22 (68.75)	32 (100)	
Socioeconomic status, n (%)				
Low low	17 (44.74)	21 (55.26)	38 (100)	0.060
Low	61 (40.67)	89 (59.33)	150 (100)	
Middle low	28 (31.11)	62 (68.89)	90 (100)	
Middle	4 (28.57)	10 (71.43)	14 (100)	
Middle high	4 (30.77)	9 (69.23)	13 (100)	
High	0 (0.00)	0 (0.00)	0 (100)	
Probable PTSD, n(%)				
No	90 (32.03)	191 (67.97)	281 (100)	< 0.001
Yes	24 (100)	0 (0.00)	24 (100)	
Alcohol Use, n(%)				
No	77 (28.73)	191 (71.27)	268 (100)	< 0.001
Yes	37 (100)	0 (0.00)	37 (100)	
Emotional abuse, n(%)				
No	78 (31.45)	170 (68.55)	248 (100)	< 0.001
Yes	36 (63.16)	21 (36.84)	57 (100)	
Physical abuse, n(%)				
No	76 (30.28)	175 (69.72)	251 (100)	< 0.001
Yes	38 (70.37)	16 (29.63)	54 (100)	
Sexual abuse, n(%)				
No	78 (30.12)	181 (69.88)	259 (100)	< 0.001
Yes	36 (78.26)	10 (21.74)	46 (100)	
Emotional neglect, n(%)				
No	79 (30.86)	177 (69.14)	256 (100)	< 0.001
Yes	35 (71.43)	14 (28.57)	49 (100)	
Physical neglect, n(%)				
No	89 (33.09)	180 (66.91)	269 (100)	< 0.001
Yes	25 (69.44)	11 (30.56)	36 (100)

**Table 2:** Severity factors in bipolar patients (N = 114)

	Cases n, (%)
No. cases	114 (100)
Bipolar disorder type	
BD I	70 (61.40)
BD II	44 (38.60)
Severity factors	
Age of onset	
< 25 years	91 (79.8)
≥ 25 years	23 (20.2)
Suicidal ideation or attempt	
Yes	40 (35.1)
No	74 (64.9)
Rapid cycling	
Yes	35 (30.7)
No	79 (69.3)
No. hospitalization per year	
≥ 3	69 (60.5)
< 3	45 (39.5)
Early age of onset + suicidal ideation or attempt	
Yes	40 (35.1)
No	74 (64.9)
Early age of onset + rapid cycling	
Yes	33 (28.9)
No	81 (71.1)
Early age of onset + ≥ 3 hospitalization per year	
Yes	59 (51.7)
No	55 (48.3)
Early age of onset + suicidal ideation or attempt+ rapid cycling	
Yes	21 (18.4)
No	93 (81.6)
Early age of onset + suicidal ideation or attempt + rapid cycling + ≥ 3 hospitalization per year	
Yes	18 (15.8)
No	96 (84.2)

### Association between Childhood Trauma and severe bipolar disorder

Disaggregated analysis for each subcategory of childhood trauma according to the factor structure of CTQ-SF were performed. A significant relationship was identified between each subcategory and the established clinical outcomes of severity illness.

#### Childhood trauma and early age of onset

A positive relationship were identified with regards to emotional abuse, physical abuse, sexual abuse, physical neglect and emotional neglect (χ^2^ = 17.40; *df* = 1; *p* < 0.001; χ^2^ = 38.35; *df* = 1; *p* < 0.00; χ^2^ = 36.49; *df* = 1; *p* < 0.001; χ^2^ = 15.83; *df* = 1; *p* < 0.001; χ^2^ = 12.51; *df* = 1; *p* < 0.001) (Table 3).

**Table 3:** Severity factors associated to childhood trauma

Severity outcomes	Childhood trauma subcategories																			
	Emotional abuse				Physical abuse				Sexual abuse				Physical neglect				Emotional neglect			
	No n, (%)	Yes n, (%)	total n, (%)	p value	No n, (%)	Yes n, (%)	Total n, (%)	p value	No n, (%)	Yes n, (%)	Total n, (%)	p value	No n, (%)	Yes n, (%)	Total n, (%)	p value	No n, (%)	Yes n, (%)	Total n, (%)	p value
Age onset																				
> 25	187 (75.40)	61 (24.60)	248 (100)	< .001	195 (77.69)	56 (22.31)	251 (100)	< .001	199 (76.83)	60 (23.17)	259 (100)	< .001	199 (73.98)	70 (26.02)	269 (100)	< .001	190 (74.22)	66 (25.78)	256 (100)	< .001
≤ 25	27 (47.37)	30 (52.63)	57 (100)		19 (35.19)	35 (64.81)	54 (100)		15 (32.61)	31 (67.39)	46 (100)		15 (41.67)	21 (58.33)	36 (100)		24 (48.98)	25 (51.02)	49 (100)	
Suicidal Ideation or Attempt																				
No	225 (90.73)	23 (9.27)	248 (100)	< .001	231 (92.03)	20 (7.97)	251 (100)	< .001	237 (91.51)	22 (8.49)	259 (100)	< .001	242 (89.96)	27 (10.04)	269 (100)	< .001	231 (90.23)	25 (9.77)	256 (100)	< .001
Yes	40 (70.18)	17 (29.82)	57 (100)		34 (62.96)	20 (37.04)	54 (100)		28 (60.87)	18 (39.13)	46 (100)		23 (63.89)	13 (36.11)	36 (100)		34 (69.39)	15 (30.61)	49 (100)	
Rapid cycling																				
No	226 (91.13)	22 (8.87)	248 (100)	.003	235 (93.63)	16 (6.37)	251 (100)	< .001	239 (92.28)	20 (7.72)	259 (100)	< .001	248 (92.19)	21 (7.81)	269 (100)	< .001	234 (91.41)	22 (8.59)	256 (100)	< .001
Yes	44 (77.19)	13 (22.81)	57 (100)		35 (64.81)	19 (35.19)	54 (100)		31 (67.39)	15 (32.61)	46 (100)		22 (61.11)	14 (38.89)	36 (100)		36 (73.47)	13 (26.53)	49 (100)	
Number hospitalization per year																				
< 3	44 (17.74)	204 (82.26)	248 (100)	< .001	37 (14.74)	214 (85.26)	251 (100)	< .001	37 (14.29)	222 (85.71)	259 (100)	< .001	48 (17.84)	221 (82.16)	269 (100)	< .001	47 (18.36)	209 (81.64)	256 (100)	< .001
≥ 3	25 (43.86)	32 (56.14)	57 (100)		32 (59.26)	22 (40.74)	54 (100)		32 (69.57)	14 (30.43)	46 (100)		21 (58.33)	15 (41.67)	36 (100)		22 (44.90)	27 (55.10)	49 (100)	

*EA* Emotional Abuse, *PA* Physical Abuse, *SA* Sexual Abuse, *PN* Physical Neglect, *EN* Emotional Neglect

#### Suicide attempt or ideation

The results showed a relationship between suicide attempt or ideation and childhood trauma subcategories for emotional abuse, physical abuse, sexual abuse, physical neglect or emotional neglect (χ^2^ = 17.17; *df* = 1; *p* < 0.001; χ^2^ = 32.95; *df* = 1; *p* < 0.001; χ^2^ = 32.17; *df* = 1; *p* < 0.001; χ^2^ = 18.94; *df* = 1; *p* < 0.001; χ^2^ = 15.68; *df* = 1; *p* < 0.001) (Table 3).

#### Rapid cycling

Bipolar patients with rapid cycling history also had a positive relationship between emotional abuse, physical abuse, sexual abuse, physical neglect, or emotional neglect (χ^2^ = 8.86; *df* = 1; *p* < 0.001; χ^2^ = 36.31; *df* = 1; *p* < 0.001; χ^2^ = 23.81; *df* = 1; *p* < 0.001; χ^2^ = 30.19; *df* = 1; *p* < 0.001; χ^2^ = 13.02; *df* = 1; *p* < 0.001) (Table 3).

#### Hospitalization per year

After dichotomizing by 3 or higher hospitalizations per year, we identified a positive relationship between emotional abuse, physical abuse, sexual abuse, physical neglect, or emotional neglect (χ^2^ = 18.06; *df* = 1; *p* < 0.001; χ^2^ = 50.31; *df* = 1; *p* < 0.001; χ^2^ = 68.19; *df* = 1; *p* < 0.001; χ^2^ = 29.73; *df* = 1; *p* < 0.001; χ^2^ = 16.54; *df* = 1; *p* < 0.001) (Table 3).

### Multivariate logistic regression models to explain severe bipolar disorder

Multivariable logistic regression analysis controlling for confounders (age, gender, marital status, socioeconomic status, probable PTSD symptoms, and positive alcohol use symptoms), described that having an emotional abuse, physical abuse, sexual abuse, and emotional neglect were strong associated with severe bipolar disorder (*OR* = 2.30; 95% CI, 1.75–3.03; *p* < 0.001; *OR* = 2.92; 95% CI, 1.54–5.53; *p* < 0.001; *OR* = 5.04; 95% CI, 4.73–5.36; *p* < 0.001; *OR* = 1.32; 95% CI, 0.93–1.87; *p* < 0.001; *OR* = 3.45; 95% CI, 2.28–5.23; *p* < 0.001, respectively) (Table 4).

**Table 4:** Multivariate logistic regression model in severe bipolar disorder

Variable	Severe bipolar disorder							*R*^*2*^
	*B*	*SE*	*OR*	95% CI		*p* value	*p* model	
Emotional abuse	0.83	0.36	2.30	1.75	3.03	< 0.001	< 0.001	0.10
Physical abuse	1.07	0.43	2.92	1.54	5.53	< 0.001	< 0.001	
Sexual abuse	1.61	0.44	5.04	4.73	5.36	< 0.001	< 0.001	
Physical neglect	0.28	0.49	1.32	0.93	1.87	0.117	< 0.001	
Emotional neglect	1.24	0.38	3.45	2.28	5.23	< 0.001	< 0.001	

A robust logistic regression model clustered by case–control groups and adjusted by age, gender, marital status, socioeconomic status

*B* regression coefficient, *SE* Standard Error, *OR* Odds Ratio, *CI* Confidence Interval, *R*^*2*^ Adjusted R-Squared

In a desegregated logistic regression analysis, emotional abuse, physical abuse, sexual abuse and physical neglect were more likely to explain the early age of onset in bipolar patients. Suicide attempt or ideation was associated to emotional abuse, physical abuse, physical neglect and emotional neglect but not with sexual abuse. Rapid cycling was associated with physical abuse, physical and emotional neglect. Physical and sexual abuse were associated with ≥ 3 hospitalization per year in bipolar patients. (Table 5).

**Table 5:** Multivariate logistic regression for clinical indicators of severity in bipolar disorder

	*B*	*SE*	*OR*	95% CI		*p* value	*p* model	*R*^2^ (McFadden)
**Early age of onset**								
Emotional abuse	0.81	0.35	2.24	1.60	3.17	< .001	< .001	0.045
Physical abuse	1.24	0.42	3.48	2.31	5.26	< .001		
Sexual abuse	1.16	0.40	3.21	1.22	8.42	0.018		
Physical neglect	0.39	0.47	0.96	0.95	0.96	< .001		
Emotional neglect	0.69	0.37	2.01	0.79	5.08	0.139		
**Suicide attempt or ideation**								
Emotional abuse	0.90	0.42	2.48	1.48	4.15	0.001	< .001	0.014
Physical abuse	0.97	0.49	2.66	1.83	3.87	< .001		
Sexual abuse	1.05	0.46	2.86	0.81	10.11	0.102		
Physical neglect	0.40	0.51	1.50	1.07	2.11	0.018		
Emotional neglect	0.91	0.43	2.48	1.15	5.33	0.020		
**Rapid cycling**								
Emotional abuse	0.58	0.46	1.80	0.84	3.81	0.126	< .001	0.016
Physical abuse	1.19	0.52	3.30	1.85	5.84	< .001		
Sexual abuse	0.80	0.49	2.24	0.75	6.62	0.145		
Physical neglect	0.85	0.51	2.34	1.51	3.62	< .001		
Emotional neglect	0.91	0.47	2.50	1.06	5.87	0.036		
**Hospitalization ≥ 3 per year**								
Emotional abuse	0.72	0.40	2.07	0.91	4.70	0.082	< .001	0.151
Physical abuse	1.26	0.46	3.54	3.17	3.96	< .001		
Sexual abuse	1.92	0.43	6.84	4.20	11.17	< .001		
Physical neglect	0.38	0.50	1.46	0.93	2.30	0.095		
Emotional neglect	0.99	0.42	2.70	0.93	7.77	0.066		

A robust logistic regression model clustered by case–control groups and adjusted by age, gender, marital status, socioeconomic status and alcohol use/abuse

*B* regression coefficient, *SE* Standard Error, *OR* Odds Ratio, *CI* Confidence Interval, *R*^*2*^ Adjusted R-Squared

## Discussion

To the best of our knowledge, this is the first case–control study that describes a higher proportion of childhood trauma in bipolar patients, and an association for childhood trauma exposure and severity of the course of illness in bipolar disorder in a low-and middle-income country in Latin-America such as Colombia. As we mentioned above, in our study design the exposures were defined according to the subcategories of the Childhood Trauma Questionnaire-Short Form defined previously for childhood trauma (30). The outcome variables were: (i) severe bipolar disorder defined as the presence of any clinical severity indicators collected (early age of onset, rapid cycling, past history of suicidal ideation or suicide attempts and three or more psychiatric unit hospitalization per year) and (ii) for each clinical indicator of severity as an outcome variable. Our principal findings are: (i) a higher prevalence of childhood trauma in the bipolar patients compared to controls, (ii) a positive relationship of childhood trauma subcategories with clinical severity indicators of course of illness such as early age of onset, rapid cycling, attempt of suicidal ideation, and three or more hospitalizations per year in mental health units, and (iii) a strong association of childhood trauma with severe bipolar disorder.

Etain et al. showed that childhood trauma was significantly more frequent in bipolar disorder patients than in controls (63 vs. 33%) (Etain et al. 2010). According to our results, the highest proportion of childhood trauma was identified in the subcategory of physical abuse with 33.33% of cases versus 8.38% of controls. In this sense, previous studies has reported that sexual abuse and physical abuse are the most frequently subcategory related to bipolar disorder patients (Aas et al. 2016b). Our findings evidenced that sexual abuse is the second more prevalent childhood trauma subcategory but has the highest association with the severity of the disease compared to the other subcategories (emotional abuse, physical abuse, physical neglect and emotional neglect) (See Table 4). A meta-analysis published by Zhang et al. (2020a) reported a prevalence of 30%, 18%, 22%, 30% and 31% of emotional abuse, physical abuse, sexual abuse, physical neglect, and emotional neglect respectively. According with the meta-analysis, we report consistently results in emotional abuse and emotional neglect (31.58 vs. 30.00%, 30.70 vs. 31.00%), however we reported higher proportions in physical abuse, sexual abuse (33.33 vs. 18.00%, 31.58 vs. 22.00%, respectively) and a lowest proportion in physical neglect (21.93 vs. 30.00%).

Another important aspect is the correlation with female gender where CT is more frequently in women than men in bipolar patients (Etain and Aas 2021; Aas et al. 2016a; Leboyer et al. 2007). In our study, the highest proportion of women reported childhood trauma was in the emotional neglect with a 74.29% of women cases compared to 25.71% in men. In physical abuse the proportion of women with bipolar disorder was higher, 73.68% than 26.32% in men. To physical neglect the proportion of women were 72.00% versus 28.00% of men. The 69.44% of cases exposure to emotional abuse were women compare to 30.56% of men, and the 66.67% of women cases reported sexual abuse compared to 33.33% of men in bipolar patient group. These findings were conforming with the previous studies reported related to sex-specific association in bipolar disorder patients with childhood trauma (Fisher and Hosang 2010; Etain et al. 2013).

Several clinical studies have measured negative clinical outcomes (Treuer and Tohen 2010; Agnew-Blais and Danese 2016) associated with severe mood and behavioral symptoms during the course of illness of bipolar disorder. Thus, bipolar patients with a higher number of recurrent episodes seem to have a more severe course of illness defined by an earlier age of onset (Leboyer et al. 2007; Garno et al. 2005; Leverich and Post 2006; Cate Carter et al. 2003; Suominen et al. 2007), rapid cycling (Galvez et al. 2014; Garno et al. 2005; Leverich and Post 2006), suicidal ideational or suicide attempt (Leboyer et al. 2007; Brown et al. 2015), and a higher number of hospitalizations (Galvez et al. 2014; Brown et al. 2015; Leverich et al. 2002; Carballo et al. 2008; Hamilton et al. 2016). As we show in the Table 3, our results identified a positive relationship among childhood trauma with the defined severity indicators of bipolar disorder. In bipolar patients, a history of childhood trauma has also been associated with an increased risk for earlier age of onset, rapid cycling, attempt or suicidal ideation, and more hospitalization in mental health units (Etain and Aas 2021). Our findings evidence a significant statistical higher proportion of earlier age of onset in emotional abuse, physical abuse, sexual abuse, physical neglect, and emotional neglect (Table 3). This result shows the importance of childhood trauma as an indicator of early age of onset in bipolar patients and are consistent with previous studies (Aas et al. 2016a; Garno et al. 2005). Statistical significance with rapid cycling, suicidal ideational or attempt and higher number of hospitalizations also shows a positive relationship with all childhood trauma subcategories. In this sense, our results with these clinical severity outcomes also have consistently with other studies across the world (Aas et al. 2016a).

Previous studies have reported that physical and sexual abuse are the strongest predictors of clinical indicators of severity in bipolar patients (Fisher and Hosang 2010; Daruy-Filho et al. 2011), however a high quality study published by Etain et al. (2013) also reported that emotional and sexual abuse are more specific risk factors for bipolar disorder compared to the other childhood trauma subcategories (Aas et al. 2016a; Etain et al. 2010). We reported a strong association of emotional abuse for earlier age of onset and suicide attempt or ideation; sexual abuse for earlier age of onset and ≥ 3 hospitalization per year; physical neglect for earlier age of onset, suicide attempt or ideation, and rapid cycling; emotional neglect for suicide attempt or ideation, and rapid cycling. The most consistent childhood trauma subcategory was physical abuse with a strong and significance association in all the clinical indicators of severity evaluated (Table 4).

Alcohol use or abuse is an important comorbidity in bipolar disorder patients and in people with trauma life events or childhood trauma. Our findings using the AUDIT tool identified a higher prevalence of alcohol use/abuse in bipolar patients (72.55%) than in controls (22.45%). Alcohol use/abuse evidenced a positive relation with physical abuse (χ^2^ = 5.76; *df* = 1; *p* = 0.016) however, there are no statistical significance with the other childhood trauma subcategories. A previous study on bipolar patients with childhood trauma exposure, also did not identify statistical significance between childhood trauma and alcohol dependence (Etain et al. 2013). Previous research has reported that earlier age of onset were more likely to have comorbid alcohol abuse or dependence as well as comorbid substance abuse or dependence (Cate Carter et al. 2003; Joslyn et al. 2016). In our study, 89.19% of cases with earlier age of onset (< 25 years old) evidenced alcohol use/abuse symptoms compared to 10.81% of the cases with the age of onset (≥ 25 years old). Although there is no statistical significance identified, this measure is important due to alcohol dependence has been reported widely in the literature in bipolar disorder (Farren et al. 2012; Di Florio et al. 2014) and childhood trauma exposure as associated comorbidity (Zhang et al. 2020b; Schwandt et al. 2013).

Latin America is recognized as one of the most violent geographic areas for young people (Moser and Van 1999). Around 58% of children in Latin America had experienced emotional, physical, or sexual abuse each year (Peetz 2011; Hillis et al. 2016). There are few studies in Colombia about the trauma experienced during childhood. A recent study with 2,080 Colombians assessed with the Early Trauma Inventory Self Report-Short Form evidenced that 99.8% suffered trauma in their early stages (Vallejo-Medina et al. 2021). There are no studies evaluating childhood trauma as a risk factor for developing mental disorders in our context, especially bipolar disorder. However, findings from the Colombian National Mental Health Survey report associations of mental disorders in the adolescent population displaced by violence in Colombia. This study shows a prevalence of any mental disorder in a displaced population of 11% compared to 7% of non-displaced adolescents (Marroquín Rivera et al. 2020). Therefore, this is the first study to report childhood trauma as exposure and its strong association among emotional, physical, and sexual abuse and physical, and emotional neglect with severe bipolar disorder in Colombia. With a higher prevalence reported by previous studies, and with the evidence generated with our results, policymakers and clinicians need to consider the possibility to screen childhood trauma in patients with mood disorders in conflict-settings zones such as Colombia. This is important to obtain better outcomes in the challenge of the treatment in this sub-group of bipolar patients as previously Leverich and Post has reported (Leverich and Post 2006).

Finally, Daruy-Filho et al. found that the quality of the published studies evaluating childhood trauma and bipolar disorder is poor (Daruy-Filho et al. 2011). They attribute the low quality of the studies to the following limitations, such as lack of standardized childhood trauma assessment, lack of use of structured clinical interview and the same diagnosis criteria (DSM vs ICD) for the diagnoses, sample sizes, and the lack of measures of the current mood states during the recruitment of the cases. In this sense, our research study has several strengths: minimized these limitations with specific inclusion criteria using a standard clinical interview for the diagnosis with the DSM-5 criteria, also we used a standardized and world-recognized childhood trauma tool to assess the exposure such as the CTQ-SF; more than 100 patients in the cases group, and at the recruitment point assessments of the current mood state with the Young Mania Rating Scale and the Bipolar Depression Rating Scale to admission as cases. Finally, we measured the Alcohol Use with the AUDIT tool to controlling possible confounders. The above considerations, follow the recommendations expressed by Daruy-Filho et al. and Etain et al. in previously published works (Etain and Aas 2021; Daruy-Filho et al. 2011) to strengthen the quality of the studies and the evidence generated. However, our study also has the following limitations: the recall bias about memories during childhood assessed with the CTQ-SF, non-nationwide recruitment with a focus population in the northern region of the country, and the use of clinical indicator reporting from the electronic health records for severe illness without standardized scales.

These findings evidenced that trauma exposure in LMICs could be a critical associated factor related to affective disorders such as bipolar disorder. Childhood trauma has been associated previously with the course of illness in bipolar patients in high-income countries, however, this study reported a strong association with severe bipolar disorder in Colombia. With a strong association between trauma exposure and the course of illness in bipolar disorder, physicians could consider screening childhood trauma or traumatic life events in bipolar patients with clinical severity indicators such as early onset, rapid cycling, suicide attempt or ideation, and higher rates of hospitalization. However, with the higher proportion of trauma exposure in conflict zones settings, the early identification of trauma exposure could alert the physician to a possible severe course of the illness.

## Conclusion

Our results evidence an important prevalence of childhood trauma in a population of bipolar patients in Colombia. There is a strong association between subcategories of childhood trauma with severe course of bipolar disorder. We should always remember that bipolar disorder is one of the most severe and lethal mental disorders, but it is also highly treatable (Grande et al. 2016; Yatham et al. 2018; Vieta et al. 2018). At this moment, being able to understand better how childhood trauma exposure might anticipate or modify the course of illness in bipolar disorder has become one of the main goals for our research group. These results should give researchers and clinicians worldwide more diagnostic and therapeutic tools to tailor better multimodal interventions for patients with a severe course of bipolar disorder focusing on childhood trauma exposure and its consequence We hope our efforts in bipolar international research help many clinicians, nurses, families, and patients to identify these and other several clinical factors that may characterize those bipolar patients with higher rates previous of childhood trauma exposure.

## Data Availability

The data that support the findings of this study are available from the corresponding author upon reasonable request.

## Abbreviations

AUDIT: Alcohol Use Disorders Identification Test
BD: Bipolar Disorder
BDRS: Bipolar Depression Rating Scale
CTQ-SF: Childhood Trauma Questionnaire Short Form
DSM-5: Diagnostic and Statistical Manual of Mental Disorder, Fifth Edition
IQR: Inter-quartile Range
LEC-5: Life Events Checklist for DSM-5
*OR*: Odds Ratio
PCL-5: Post-traumatic Stress Disorder Checklist for DSM-5
PTSD: Post-traumatic Stress Disorder
YMRS: Young Mania Rating Scale
